# Serum insulin-like growth factor binding protein 2 is associated with hepatic steatosis in adults with metabolic dysfunction-associated steatotic liver disease

**DOI:** 10.1530/EC-25-0285

**Published:** 2025-07-17

**Authors:** Ziwei Wang, Hongyan Wu, Hsiuying Fu, Yingying Xue, Lixuan Shen, Jingyu Zhu, Ziwei Zhu, Xizhong Yu, Ruonan Zhou, Wenbin Shang

**Affiliations:** ^1^ Department of Endocrinology, Jiangsu Province Hospital of Chinese Medicine, The Affiliated Hospital of Nanjing University of Chinese Medicine, Nanjing, China; ^2^ Key Laboratory for Metabolic Diseases in Chinese Medicine, Nanjing University of Chinese Medicine, Nanjing, China

**Keywords:** metabolic dysfunction-associated steatotic liver disease, insulin-like growth factor binding protein 2, liver fibrosis, hepatic steatosis

## Abstract

**Objective:**

To evaluate the association between serum insulin-like growth factor binding protein 2 (IGFBP2) and the degree of hepatic steatosis in patients with MASLD.

**Methods:**

A total of 347 patients with metabolic dysfunction-associated steatotic liver disease (MASLD) were enrolled, adhering to the inclusion criteria. The controlled attenuation parameter (CAP) and liver stiffness measurement (LSM) were measured by FibroScan, while serum IGFBP2 levels were quantified by ELISA. Hepatic IGFBP2 mRNA expression data were obtained from the Gene Expression Omnibus database (GEO database). Levels of serum IGFBP2 and hepatic IGFBP2 mRNA between healthy controls and MASLD patients were separately compared. Correlation analyses were performed to evaluate the relationships between CAP and IGFBP2.

**Results:**

Both the serum IGFBP2 levels and the hepatic IGFBP2 mRNA expression were significantly higher in patients with MASLD compared with healthy individuals. In patients with MASLD, the serum IGFBP2 level showed an inverse correlation with CAP values (*r* = −0.133, *P* < 0.05) and was identified as an independent determinant of hepatic steatosis (*β* = −0.104, *P* < 0.05), while no significant association was observed between LSM and IGFBP2 (*P* > 0.05).

**Conclusion:**

In patients with MASLD, IGFBP2 may exert a protective effect against hepatic steatosis progression but appears to play a negligible role in fibrogenesis.

## Introduction

The prevalence of metabolic dysfunction-associated steatotic liver disease (MASLD) has risen sharply in recent decades, mirroring the global surge in obesity, type 2 diabetes, and metabolic syndrome. Defined as hepatic steatosis occurring in the presence of metabolic dysregulation, such as insulin resistance, hyperlipidemia, or hypertension, MASLD represents a paradigm shift from the traditional ‘non-alcoholic fatty liver disease’ (NAFLD) framework by emphasizing metabolic drivers rather than alcohol exclusion (1). This condition not only ranks as the leading cause of chronic liver disease worldwide but also confers a heightened risk for progressive liver outcomes, including inflammation, fibrosis, cirrhosis, and hepatocellular carcinoma (2). Critically, MASLD’s systemic impact extends beyond the liver, manifesting as strong associations with cardiovascular disease, chronic kidney dysfunction, and extrahepatic malignancies (3, 4, 5). Despite the escalating clinical burden of MASLD, the pathophysiological mechanisms underpinning MASLD remain incompletely understood (6). Equally problematic are the absence of validated noninvasive intervention biomarkers and the limitation of therapeutic interventions (7).

Insulin-like growth factor binding protein 2 (IGFBP2) is a binding protein of insulin-like growth factors (IGFs) and is widely expressed in various cell types, such as adipocytes, hepatocytes, and neurons. Recent research has consistently identified IGFBP2 as a pivotal regulator of lipid metabolism. IGFBP2 is one of the most abundantly secreted proteins by white pre-adipocytes during the progress of adipogenesis within the IGFBP family (8, 9, 10). It is reported that IGFBP2 regulates the activity of IGF1 by competitively blocking its interaction with the IGF receptor, thereby inhibiting the activation of downstream signaling pathways, such as phosphatidylinositol 3-kinase (PI3K)/AKT and mitogen-activated protein kinases (MAPK)/extracellular signal-regulated kinase (ERK), and then restricting lipid accumulation in several cell types (11, 12). Besides, IGFBP2 also suppresses the differentiation of pre-adipocytes via its heparin-binding domain and by interacting with other factors, such as leptin, as a cytokine (13, 14).

Clues from previous studies suggest that IGFBP2 may play a role in the pathology of MASLD. Initial studies identified increased DNA methylation and decreased mRNA expression of IGFBP2 in visceral adipose tissue (VAT) of obese patients (15). Furthermore, in a small cohort of patients with obesity, it was observed that the plasma level of IGFBP2 is inversely associated with the hepatic fat fraction (16, 17). Later, a study reported that circulating IGFBP2 levels were inversely associated with the incidence of MASLD (18). However, the relationship between circulating IGFBP2 and the severity of hepatic steatosis in patients with MASLD remains unknown.

In this study, we explored the correlation between serum IGFBP2 levels and the severity of hepatic steatosis measured by the controlled attenuation parameter (CAP) in adult patients with MASLD, aiming to provide new insights for the development of potential strategies for the diagnosis and treatment of MASLD.

## Methods

### Study design

The study included 347 patients from the Affiliated Hospital of Nanjing University of Chinese Medicine, Nanjing, Jiangsu, China. Inclusion criteria were adults meeting the diagnostic criteria for MASLD, and in this study, hepatic steatosis is delineated by a CAP ≥248 dB/m measured by FibroScan (1). Furthermore, cases with MASLD and increased alcohol intake (MetALD; alcohol intake >30 g/day for men and >20 g/day for women) were excluded. Individuals with viral hepatitis, drug-induced liver injury, or other causes of liver disease, as well as those with severe cardiovascular, cerebrovascular, or other systemic diseases, were also excluded. In addition, 31 healthy individuals from the same hospital’s health examination center were enrolled as controls, with BMI <24 kg/m^2^ and normal ranges for liver function, blood glucose, and lipid profiles. Those taking antidiabetic or lipid-lowering medications were excluded. Informed consent was obtained from all participants before their inclusion in the study. The STROBE (Strengthening the Reporting of Observational Studies in Epidemiology) guidelines were followed for reporting the study.

### Measurement of body composition

The CAP and LSM indicators were quantitatively assessed by FibroScan. The InBody 770 was used to measure body composition parameters such as height, weight, waist circumference (WC), waist-to-hip ratio (WHR), visceral fat area (VFA), and body fat mass (BFM).

CAP thresholds: ≥248 dB/m = significant steatosis; 248–268 dB/m = moderate-severe steatosis; ≥294 dB/m = severe steatosis. LSM thresholds: 8–12 kPa = significant fibrosis (≥F2); >12 kPa = advanced fibrosis (≥F3); ≥20 kPa = cirrhosis (F4); <8 kPa = excludes advanced fibrosis (19).

### Measurement of serum biomarkers

Blood samples were collected from the antecubital vein after an 8 h overnight fast. Circulating levels of alanine transaminase (ALT), aspartate aminotransferase (AST), gamma-glutamyl transferase (GGT), fasting plasma glucose (FPG), fasting insulin (FIN), uric acid (UA), and lipid profile were quantified. Based on serum biomarkers, HOMA models (HOMA-IR, HOMA-β), liver fibrosis indices (FIB-4, APRI), and liver steatosis diagnostic models (FLI, ZJU) were applied. The study utilized residual serum samples from patients’ routine medical examinations for subsequent cytokine profiling and experimental analyses.

Serum IGFBP2 levels were determined by ELISA (Human IGFBP-2 ELISA Kit, Elabscience, China), according to the manufacturer’s instructions. The detection limit was 0.16 ng/mL. The inter-assay coefficient of variability was <10%.

### Acquisition of MASLD dataset

The datasets GSE63067 and GSE48452 were obtained from the Gene Expression Omnibus database (GEO data base; https://www.ncbi.nlm.nih.gov/geo/), which include data from 48 healthy individuals and 43 MASLD patients. The hepatic IGFBP2 mRNA expression was analyzed.

### Statistical analysis

For normally distributed continuous variables, data were reported as means ± SD. Independent sample *t*-tests were used for comparisons between two groups, and one-way ANOVA was used for comparisons among multiple groups. For data not normally distributed, variables are described using median (interquartile range) (M (P25, P75)), with the Mann–Whitney test used for two-group comparisons and the Kruskal–Wallis test for multiple group comparisons. Correlation was assessed using Pearson correlation and partial correlation analysis. Multivariate linear regression analysis was used to quantify the independent contribution of IGFBP2 to CAP. A *P* value <0.05 was considered significant.

## Results

### Elevated serum IGFBP2 concentrations and hepatic IGFBP2 mRNA expression were observed in patients with MASLD compared to healthy controls

Among 347 MASLD participants, the median BMI in the overall cohort was 33.20 kg/m^2^ (IQR 30.90–36.20), the mean CAP value was 328.85 dB/m, and the median serum IGFBP2 level was 271.48 ng/mL (IQR 204.68–395.64). Besides, the median LSM in the overall cohort was 7.10 kPa (IQR 5.70–9.90), indicating that enrolled participants predominantly exhibited mild-to-significant fibrosis (METAVIR F0–F2 stages). Detailed results are shown in Table 1.

**Table 1:** Basic clinicopathological information of 347 patients.

Characteristics	Group		
	Overall, *n* = 347 (100%)	Male, *n* = 184 (53%)	Female, *n* = 163 (47%)
Age (yrs)	30.00 (25.00, 34.00)	28.00 (25.00, 33.00)	31.00 (25.00, 36.00)
BMI (kg/m^2^)	33.20 (30.90, 36.20)	33.75 (31.50, 37.00)	32.30 (30.50, 34.60)
WC (cm)	108.90 (102.20, 118.50)	114.90 (106.93, 122.18)	104.30 (98.30, 110.60)
WHR (%)	0.99 (0.95, 1.03)	1.00 (0.97, 1.05)	0.97 (0.94, 1.00)
VFA (cm^2^)	180.50 (152.05, 204.55)	173.85 (142.78, 201.33)	184.50 (161.10, 210.90)
Weight (kg)	93.20 (83.50, 105.45)	102.75 (94.20, 113.10)	83.80 (77.10, 91.00)
Height (m)	167.67 ± 8.79	174.04 ± 6.16	160.70 ± 5.29
BFM (kg)	36.80 (32.25, 42.85)	37.05 (32.13, 43.58)	36.60 (32.60, 42.60)
CAP (Db/m)	328.85 ± 34.77	339.94 ± 31.95	315.26 ± 33.21
LSM (kPa)	7.10 (5.70, 9.90)	8.15 (6.40, 11.08)	6.20 (5.00, 8.30)
IGFBP2 (ng/mL)	271.48 (204.68, 395.64)	269.30 (209.86, 416.74)	273.21 (193.68, 372.11)
ALT (U/L)	42.00 (25.00, 74.00)	57.00 (35.25, 87.75)	28.00 (18.00, 48.00)
AST (U/L)	25.00 (19.00, 38.00)	30.00 (22.25, 43.00)	20.00 (17.00, 29.00)
GGT (U/L)	40.00 (28.00, 66.00)	56.00 (39.00, 82.00)	29.00 (22.00, 40.00)
TC (mmol/L)	4.99 (4.52, 5.76)	5.01 (4.57, 5.84)	4.97 (4.44, 5.66)
TG (mmol/L)	1.73 (1.26, 2.41)	2.01 (1.45, 2.95)	1.44 (1.11, 1.99)
HDL-C (mmol/L)	1.15 (1.02, 1.30)	1.13 (1.00, 1.27)	1.16 (1.04, 1.33)
LDL-C (mmol/L)	3.09 ± 0.73	3.15 ± 0.79	3.03 ± 0.65
FPG (mmol/L)	5.34 (5.01, 5.75)	5.31 (4.98, 5.69)	5.37 (5.05, 5.81)
FINS (Mu/L)	16.05 (11.20, 24.06)	17.44 (12.51, 25.42)	14.87 (10.36, 21.49)
HOMA-IR	3.86 (2.69, 5.84)	4.11 (2.96, 6.12)	3.52 (2.51, 5.37)
HOMA-β	181.79 (120.69, 253.52)	200.41 (133.72, 290.23)	158.57 (114.71, 232.13)
UA (μmol/L)	420.00 (350.00, 504.00)	468.00 (406.50, 535.75)	356.00 (318.00, 427.00)

*Note*: Data are expressed as the mean ± SD, median (IQR), or number (%). BMI, body mass index; CAP, controlled attenuation parameter; LSM, liver stiffness measurement; IGFBP2, insulin-like growth factor binding protein 2; ALT, alanine transaminase; AST, aspartate aminotransferase; GGT, gamma-glutamyl transferase; TC, total cholesterol; TG, triglycerides; HDL-C, high-density lipoprotein cholesterol; LDL-C, low-density lipoprotein cholesterol; FPG, fasting plasma glucose; FIN, fasting insulin; HOMA-IR, homeostasis model assessment of insulin resistance; HOMA-IS, homeostasis model assessment of insulin sensitivity; UA, uric acid.

**Figure 1:**
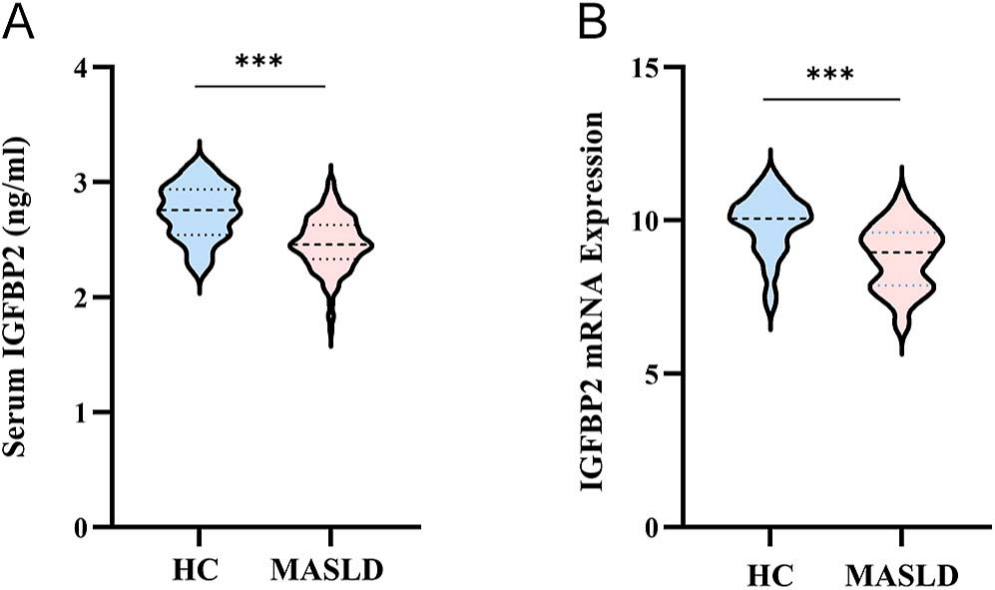
Comparative analysis of serum IGFBP2 and hepatic IGFBP2 mRNA expression between healthy controls and MASLD patients. (A) Comparison of serum IGFBP2 levels between MASLD and HC groups. (B) Differential expression of hepatic IGFBP2 mRNA between MASLD and HC groups using GSE63067 and GSE48452 database. Statistical analysis using *t*-tests demonstrated significantly reduced IGFBP2 levels in MASLD patients. The parameter IGFBP2, with a skewed distribution, underwent log(x) transformation to achieve a normal distribution before analysis. ***P < 0.001.

As shown in Fig. 1 and Supplementary Fig. S1 (see section on Supplementary materials given at the end of the article), compared with the healthy control (HC) group, the MASLD group showed a profoundly decreased serum IGFBP2 level (Fig. 1A) and BMI (Supplementary Fig. S1B), with no significant difference in age (Supplementary Fig. S1A).

Moreover, we did a reanalysis on the hepatic transcriptomics from the GEO database (GSE63067 and GSE48452), and the results showed that the MASLD patients had significantly lower IGFBP2 mRNA levels than the healthy individuals (*P* < 0.05) (Fig. 1B).

### The serum IGFBP2 level is an independent predictor of the degree of hepatic steatosis in patients with MASLD

Considering that IGFBP2 is a hepatokine, which shows strong effects on the regulation of lipid metabolism, we estimated the degree of hepatic steatosis in patients with MASLD using CAP value from FibroScan and conducted a correlation analysis to investigate whether the serum level of IGFBP2 is associated with the degree of hepatic steatosis in patients with MASLD.

As shown in Table 2, a significant inverse association between the serum IGFBP2 level and the CAP was observed (*r* = −0.133, *P* < 0.05). We also observed a significant correlation between the CAP value and the ALT, AST, GGT, TG, FIN, and HOMA-IR in patients with MASLD (*P* < 0.05).

**Table 2:** The correlation of CAP with clinical indicators in patients with MASLD.

	Model 1		Model 2	
	*r* value	*P* value	*r* value	*P* value
IGFBP2 (ng/mL)	−0.101	0.061	−0.133	0.014*
BMI (kg/m^2^)	0.430	<0.001*	0.060	0.753
WHR (%)	0.326	<0.001*	0.006	0.910
VFA (cm^2^)	0.235	<0.001*	−0.067	0.213
BFM (kg)	0.363	<0.001*	−0.068	0.206
LSM (kPa)	0.334	<0.001*	0.103	0.056
ALT (U/L)	0.436	<0.001*	0.310	<0.001*
AST (U/L)	0.315	<0.001*	0.198	<0.001*
GGT (U/L)	0.312	<0.001*	0.140	0.009*
TC (mmol/L)	−0.028	0.599	−0.021	0.704
TG (mmol/L)	0.205	<0.001*	0.131	0.015*
HDL-C (mmol/L)	−0.105	0.050	−0.044	0.484
LDL-C (mmol/L)	−0.10	0.849	−0.010	0.903
FPG (mmol/L)	0.093	0.084	0.067	0.216
HbA1c (%)	0.163	0.002*	0.099	0.068
FINS (mU/L)	0.296	<0.001*	0.113	0.037*
HOMA-IR	0.293	<0.001*	0.118	0.028*
HOMA-β	0.229	<0.001*	0.059	0.275
UA (umol/L)	0.235	<0.001*	0.050	0.357

*Note:* Correlation analyses were conducted to examine the association between CAP and possible factors. Model 1: no variable adjustment (Pearson); Model 2: adjusted for age, height, and weight based on Model 1 (Partial). The parameters IGFBP2, VFA, BMI, WC, BFM, LSM, AST, ALT, GGT, TC, TG, HDL-C, FPG, HbA1c, FINS, HOMA-IR, HOMA-β, and UA with a skewed distribution, underwent log(x) transformation to achieve a normal distribution before analysis.

* Significance, *P* < 0.05.

To further elucidate the relationship between IGFBP2 and CAP, a multiple linear regression analysis was conducted. As shown in Table 3, IGFBP2 (*β* = −0.104, *P* < 0.05), along with BFM (*β* = 0.327, *P* < 0.05), ALT (*β* = 0.135, *P* < 0.05), and TG (*β* = 0.161, *P* < 0.05), were independent predictors of the CAP value in patients with MASLD.

**Table 3:** Risk factors of CAP in patients with MASLD.

Variables	*B*	*β*	*P* value	Adjusted *R*^2^	*F*
IGFBP2 (ng/mL)	−15.809	−0.104	0.031*	0.213	14.414*
BFM (kg)	1.203	0.327	<0.001*		
ALT (U/L)	0.062	0.135	0.008*		
TG (mmol/L)	3.489	0.161	0.001*		
HDL-C (mmol/L)	−10.731	−0.063	0.196		
FINS (mU/L)	0.208	0.063	0.244		
UA (umol/L)	0.029	0.092	0.069		

*Note:* Multiple linear regression analysis between CAP and possible variables. The parameter IGFBP2 was log-transformed before analysis.

* *P* < 0.05.

To mitigate potential confounding effects of severe obesity on CAP measurement accuracy, we conducted a subgroup analysis in participants with BMI <40 kg/m^2^ (*n* = 316), which yielded consistent results with the primary cohort (Supplementary Tables S1 and S2).

### The serum level of IGFBP2 is not correlated with the degree of hepatic fibrosis in patients with MASLD

Hepatic fibrosis is a critical determinant of long-term prognosis in MASLD patients, as it directly influences the risk of progression to cirrhosis, liver failure, and hepatocellular carcinoma. Thus, we also evaluated the association between the serum level of IGFBP2 and the LSM value (a hepatic fibrosis predictor from FibroScan).

Surprisingly, the results showed that IGFBP2 was not significantly correlated with LSM (*P* > 0.05) (Table 4). The same conclusion was corroborated in a subgroup analysis excluding individuals with severe obesity (Supplementary Table S1), indicating that IGFBP2 is unlikely to be a major contributor to the hepatic fibrosis process. In addition, the analysis revealed that independent predictors of LSM include ALT, AST, GGT, HbA1c, FINS, HOMA-IR, HOMA-β, and UA.

**Table 4:** Associations of serum IGFBP2, body composition, and metabolic variables with LSM.

	Model 1		Model 2	
	*r* value	*P* value	*r* value	*P* value
IGFBP2 (ng/mL)	0.059	0.272	0.069	0.202
BMI (kg/m^2^)	0.441	<0.001*	−0.056	0.300
WHR (%)	0.290	<0.001*	−0.013	0.807
VFA (cm^2^)	0.253	<0.001*	−0.082	0.129
BFM (kg)	0.380	<0.001*	−0.083	0.122
ALT (U/L)	0.380	<0.001*	0.290	<0.001*
AST (U/L)	0.435	<0.001*	0.329	<0.001*
GGT (U/L)	0.385	<0.001*	0.267	<0.001*
TC (mmol/L)	0.062	0.250	0.101	0.062
TG (mmol/L)	0.149	0.005*	0.096	0.074
HDL-C (mmol/L)	−0.115	0.032*	−0.061	0.256
LDL-C (mmol/L)	0.086	0.112	0.108	0.044*
FPG (mmol/L)	0.168	0.002*	0.173	0.001*
HbA1c (%)	0.237	<0.001*	0.195	<0.001*
FINS (mU/L)	0.395	<0.001*	0.231	<0.001*
HOMA-IR	0.401	<0.001*	0.251	<0.001*
HOMA-β	0.297	<0.001*	0.131	0.015*
UA (umol/L)	0.335	<0.001*	0.196	<0.001*

*Note:* Correlation analyses were conducted to examine the association between LSM and possible factors. Model 1: no variable adjustment (Pearson); Model 2: adjusted for age, height, and weight based on Model 1 (Partial). The parameters LSM, IGFBP2, VFA, BMI, WC, BFM, AST, ALT, GGT, TC, TG, HDL-C, FPG, HbA1c, FINS, HOMA-IR, HOMA-β, and UA, with a skewed distribution, underwent log(x) transformation to achieve a normal distribution before analysis.

* Significance, *P* < 0.05.

## Discussion

Although major breakthroughs have been made in the mechanisms underlying MASLD over the last decades, the diagnosis and treatment of MASLD remain huge challenges in clinical practice. Our study demonstrates that the decreased serum level of IGFBP2 is an independent contributor to hepatic steatosis rather than hepatic fibrosis in patients with MASLD. These results suggest that IGFBP2 plays an important role in the pathology of MASLD and provide novel perspectives in developing new diagnostic, as well as therapeutic, strategies for MASLD.

There are currently several proposed mechanisms by which IGFBP2 affects hepatic lipid deposition. An integrated bioinformatics and experimental study found that hepatic IGFBP2 expression was inversely associated with steatosis and serum ALT/AST levels. Moreover, IGFBP2 overexpression attenuated oleic acid (OA)-induced TG accumulation in HepG2 cells (20). IGFBP2 has also been demonstrated to activate the AMP-activated protein kinase (AMPK) signaling pathway, resulting in intracellular translocation of glucose transporter 4 (GLUT4), stimulating glucose uptake, improving insulin resistance, and subsequently impacting lipid deposition in hepatocytes (21). A study previously found robust statistical interactions between IGFBP2 levels and VAT for indices of plasma glucose-insulin homeostasis, suggesting that IGFBP2 may indirectly contribute to a low liver fat phenotype by enhancing insulin sensitivity despite the presence of visceral obesity (17). IGFBP2 may also influence hepatic steatosis through its interaction with IGF1. Previous research has established an association between circulating IGF1 levels and MASLD, suggesting that IGFBP2 could modulate the presence or severity of MASLD via IGF1 binding (22). Subsequent studies have shown that IGFBP2 blunts the stimulation of *de novo* lipogenesis by IGF1 in hepatocytes from healthy mice or mouse models with fatty liver disease, suggesting that lower levels of IGFBP2 may exacerbate the development of MASLD (23). In addition, IGFBP2 can interact with epidermal growth factor receptor via the sequence of 233e257 amino acids, inhibiting the downstream signal transducer and activator of transcription signaling pathway, reducing the promoter activity of *srebf1*, and downregulating the expression of several genes involved in lipogenesis, thereby alleviating hepatic steatosis (24). IGFBP2 has also been shown to engage integrins through its Arg-Gly-Asp (RGD) domain, thereby promoting the expression of heat shock protein 90 (Hsp90). This interaction activates the Raf-dependent ERK signaling cascade, leading to upregulation of β-catenin and subsequent inhibition of adipogenesis (25). Recent studies have demonstrated that suppression of IGFBP2 expression mitigates FGF1-induced activation of AMPK, supporting a critical role for IGFBP2 in mediating the therapeutic effects of FGF1 on obesity-associated hepatic steatosis (26).

Interestingly, we did not observe a significant correlation between the serum level of IGFBP2 and the hepatic fibrosis indicator LSM in patients with MASLD in this research. In a previous study, it was found that the circulating level of IGFBP2 was elevated specifically in F4 stage fibrosis patients with chronic hepatitis C (CHC) but showed no significant changes in F0–F3 stages (27). A possible reason for this contradiction is that in our study, which primarily enrolled MASLD patients with mild-to-significant fibrosis (F0–F2), confirmed no significant correlation between IGFBP2 and LSM, suggesting a limited role for IGFBP2 in early-stage fibrosis. This observation may reflect stage-specific involvement of IGFBP2 in fibrogenic signaling pathways and oxidative stress responses. Early fibrosis is predominantly driven by hepatic stellate cell activation mediated by TGF-β and PDGF, with no evidence of direct IGFBP2 involvement in these canonical pathways. In contrast, at advanced stages (F4), IGFBP2 expression may be induced via non-canonical mechanisms, such as the PI3K/AKT/mTOR pathway (28). Furthermore, chronic hypoxia and mitochondrial dysfunction associated with progressive fibrosis may stimulate IGFBP2 expression through the hypoxia-inducible factor-1 (HIF-1α)-IGFBP2 axis (29), while such induction is absent in early-stage fibrosis due to lower oxidative stress levels.

Nevertheless, it should be acknowledged that the current study has some limitations, such as the reliance on FibroScan measurement of CAP as the diagnostic criterion for fatty liver. Although CAP is recognized for having decreased accuracy in certain conditions and still lacks a universally accepted cutoff value, it is accurate in grading fatty infiltration (30, 31, 32). Furthermore, our study confirmed that the relationship between IGFBP2 and CAP remained consistent across both the overall cohort and the subgroup excluding individuals with severe obesity. Therefore, the study deems the use of CAP to be a valuable reference. In addition, the age range of the participants included in this study is approximately 20–60 years, and the sample size is relatively small, so our results need to be validated in independent cohorts. Larger-scale studies are needed to confirm these results in a more diverse MASLD cohort and to assess the clinical utility of IGFBP2 as a biomarker or therapeutic target.

## Conclusion

In conclusion, our findings revealed that serum IGFBP2 is inversely associated with CAP and independently contributes to CAP variation, whereas no correlation was found between IGFBP2 and LSM. This suggests that IGFBP2 may be involved in mitigating hepatic steatosis but does not appear to play a major role in liver fibrosis progression.

## Supplementary materials



## Declaration of interest

The authors declare that there is no conflict of interest that could be perceived as prejudicing the impartiality of the work reported.

## Funding

This work was supported by the National Natural Science Foundation of Chinahttps://doi.org/10.13039/501100001809 Grant Awards (82474139) and the Postgraduate Research & Practice Innovation Program of Jiangsu Province (SJCX24_0982). The Priority Academic Program Development of Jiangsu Higher Education Institutionshttps://doi.org/10.13039/501100012246 (035062005006-21).

## Author contribution statement

Ziwei Wang contributed to the writing of the original draft, formal analysis, methodology, software, visualization, funding acquisition. Hongyan Wu was responsible for validation, investigation, and methodology. Xiuying Fu and Lixuan Shen contributed to investigation and methodology. Jingyu Zhu, Ziwei Zhu, Yingying Xiang, Yue Cao, and Xizhong Yu were involved in validation and methodology. Ruonan Zhou contributed to methodology, writing of the review and editing, and supervision. Wenbin Shang was responsible for conceptualization, writing of the review and editing, resources, supervision, project administration, and funding acquisition.

## Ethics declaration

This study was approved by the Ethics Committee of the Affiliated Hospital of Nanjing University of Chinese Medicine (project number: 2024NL-227-02). All experiments were performed in accordance with relevant guidelines and regulations.

## References

[1] Rinella ME, Lazarus JV, Ratziu V, et al. A multisociety Delphi consensus statement on new fatty liver disease nomenclature. Hepatology 2023 78 1966–1986. (10.1097/HEP.0000000000000520) 37363821 PMC10653297

[2] Loomba R, Wong R, Fraysse J, et al. Nonalcoholic fatty liver disease progression rates to cirrhosis and progression of cirrhosis to decompensation and mortality: a real world analysis of Medicare data. Aliment Pharmacol Ther 2020 51 1149–1159. (10.1111/apt.15679) 32372515

[3] Byrne CD & Targher G. NAFLD as a driver of chronic kidney disease. J Hepatol 2020 72 785–801. (10.1016/j.jhep.2020.01.013) 32059982

[4] Park MK, Hur MH, Moon HS, et al. Extrahepatic malignancies in metabolic dysfunction-associated fatty liver disease: a nationwide cohort study. Liver Int 2024 44 799–810. (10.1111/liv.15832) 38230848

[5] Lonardo A, Nascimbeni F, Mantovani A, et al. Hypertension, diabetes, atherosclerosis and NASH: cause or consequence? J Hepatol 2018 68 335–352. (10.1016/j.jhep.2017.09.021) 29122390

[6] Peiseler M, Schwabe R, Hampe J, et al. Immune mechanisms linking metabolic injury to inflammation and fibrosis in fatty liver disease – novel insights into cellular communication circuits. J Hepatol 2022 77 1136–1160. (10.1016/j.jhep.2022.06.012) 35750137

[7] Abdelhameed F, Kite C, Lagojda L, et al. Non-invasive scores and serum biomarkers for fatty liver in the era of metabolic dysfunction-associated steatotic liver disease (MASLD): a comprehensive review from NAFLD to MAFLD and MASLD. Curr Obes Rep 2024 13 510–531. (10.1007/s13679-024-00574-z) 38809396 PMC11306269

[8] Hjortebjerg R, Kristiansen MR, Brandslund I, et al. Associations between insulin-like growth factor binding protein-2 and insulin sensitivity, metformin, and mortality in persons with T2D. Diabetes Res Clin Pract 2023 205 110977. (10.1016/j.diabres.2023.110977) 37890435

[9] Carter S, Li Z, Lemieux I, et al. Circulating IGFBP-2 levels are incrementally linked to correlates of the metabolic syndrome and independently associated with VLDL triglycerides. Atherosclerosis 2014 237 645–651. (10.1016/j.atherosclerosis.2014.09.022) 25463100

[10] van den Beld AW, Blum WF, Brugts MP, et al. High IGFBP2 levels are not only associated with a better metabolic risk profile but also with increased mortality in elderly men. Eur J Endocrinol 2012 167 111–117. (10.1530/eje-12-0160) 22555360

[11] Liu Y, Nelson MV, Bailey C, et al. Correction: targeting the HIF-1α-IGFBP2 axis therapeutically reduces IGF1-AKT signaling and blocks the growth and metastasis of relapsed anaplastic Wilms tumor. Oncogene 2022 41 1383. (10.1038/s41388-021-02042-7) 35046533

[12] Liu X, Chen S & Zhang L. Downregulated microRNA-130b-5p prevents lipid accumulation and insulin resistance in a murine model of nonalcoholic fatty liver disease. Am J Physiol Endocrinol Metab 2020 319 E34–e42. (10.1152/ajpendo.00528.2019) 32228319

[13] Hedbacker K, Birsoy K, Wysocki RW, et al. Antidiabetic effects of IGFBP2, a leptin-regulated gene. Cell Metab 2010 11 11–22. (10.1016/j.cmet.2009.11.007) 20074524

[14] Wang W, Ye J, Xu L, et al. The effects of IGF-1 and IGFBP-2 treatments on the atherosclerosis in the aorta and the coronary arteries of the high cholesterol diet-fed rabbits. Int Immunopharmacol 2024 127 111409. (10.1016/j.intimp.2023.111409) 38118312

[15] Zhang X, Gu HF, Frystyk J, et al. Analyses of IGFBP2 DNA methylation and mRNA expression in visceral and subcutaneous adipose tissues of obese subjects. Growth Hormone IGF Res 2019 45 31–36. (10.1016/j.ghir.2019.03.002) 30921666

[16] Rauzier C, Chartrand DJ, Alméras N, et al. Associations of insulin-like growth factor binding protein-2 with metabolic profile and hepatic fat deposition in asymptomatic men and women. Am J Physiol Endocrinol Metab 2023 325 E99–e105. (10.1152/ajpendo.00108.2023) 37285597

[17] Rauzier C, Chartrand DJ, Alméras N, et al. Plasma IGFBP-2 levels reveal heterogeneity in hepatic fat content in adults with excess visceral adiposity. Front Endocrinol 2023 14 1222101. (10.3389/fendo.2023.1222101) PMC10579942 37854178

[18] Yang J, Zhou W, Wu Y, et al. Circulating IGFBP-2 levels are inversely associated with the incidence of nonalcoholic fatty liver disease: a cohort study. J Int Med Res 2020 48 300060520935219. (10.1177/0300060520935219) 32762395 PMC7707858

[19] Mózes FE, Lee JA, Selvaraj EA, et al. Diagnostic accuracy of non-invasive tests for advanced fibrosis in patients with NAFLD: an individual patient data meta-analysis. Gut 2022 71 1006–1019. (10.1136/gutjnl-2021-324243) 34001645 PMC8995830

[20] Chen X, Tang Y, Chen S, et al. IGFBP-2 as a biomarker in NAFLD improves hepatic steatosis: an integrated bioinformatics and experimental study. Endocr Connect 2021 10 1315–1325. (10.1530/EC-21-0353) 34524971 PMC8562889

[21] Assefa B, Mahmoud AM, Pfeiffer AFH, et al. Insulin-like growth factor (IGF) binding protein-2, independently of IGF-1, induces GLUT-4 translocation and glucose uptake in 3T3-L1 adipocytes. Oxid Med Cell Longev 2017 2017 3035184. (10.1155/2017/3035184) 29422987 PMC5750484

[22] Runchey SS, Boyko EJ, Ioannou GN, et al. Relationship between serum circulating insulin-like growth factor-1 and liver fat in the United States. J Gastroenterol Hepatol 2014 29 589–596. (10.1111/jgh.12437) 24716226 PMC3982202

[23] Fahlbusch P, Knebel B, Hörbelt T, et al. Physiological disturbance in fatty liver energy metabolism converges on IGFBP2 abundance and regulation in mice and men. Int J Mol Sci 2020 21 4144. (10.3390/ijms21114144) 32532003 PMC7312731

[24] Zhai T, Cai L, Jia X, et al. IGFBP2 functions as an endogenous protector against hepatic steatosis via suppression of the EGFR-STAT3 pathway. Mol Metabol 2024 89 102026. (10.1016/j.molmet.2024.102026) PMC11474195 39299533

[25] Volloch V & Olsen BR. Why cellular stress suppresses adipogenesis in skeletal tissue, but is ineffective in adipose tissue: control of mesenchymal cell differentiation via integrin binding sites in extracellular matrices. Matrix Biol 2013 32 365–371. (10.1016/j.matbio.2013.06.001) 23792045 PMC3858580

[26] Wang J, Zhang F, Yang W, et al. FGF1 ameliorates obesity-associated hepatic steatosis by reversing IGFBP2 hypermethylation. FASEB J 2023 37 e22881. (10.1096/fj.202201950R) 36934380 PMC11977529

[27] Martínez-Castillo M, Rosique-Oramas D, Medina-Avila Z, et al. Differential production of insulin-like growth factor-binding proteins in liver fibrosis progression. Mol Cell Biochem 2020 469 65–75. (10.1007/s11010-020-03728-4) 32301061

[28] Mireuta M, Darnel A & Pollak M. IGFBP-2 expression in MCF-7 cells is regulated by the PI3K/AKT/mTOR pathway through Sp1-induced increase in transcription. Growth Factors 2010 28 243–255. (10.3109/08977191003745472) 20370577

[29] Liu Y, Nelson MV, Bailey C, et al. Targeting the HIF-1α-IGFBP2 axis therapeutically reduces IGF1-AKT signaling and blocks the growth and metastasis of relapsed anaplastic Wilms tumor. Oncogene 2021 40 4809–4819. (10.1038/s41388-021-01907-1) 34155347 PMC8319145

[30] Eddowes PJ, Sasso M, Allison M, et al. Accuracy of FibroScan controlled attenuation parameter and liver stiffness measurement in assessing steatosis and fibrosis in patients with nonalcoholic fatty liver disease. Gastroenterology 2019 156 1717–1730. (10.1053/j.gastro.2019.01.042) 30689971

[31] Wong VW, Petta S, Hiriart JB, et al. Validity criteria for the diagnosis of fatty liver by M probe-based controlled attenuation parameter. J Hepatol 2017 67 577–584. (10.1016/j.jhep.2017.05.005) 28506907

[32] Rinella ME, Neuschwander-Tetri BA, Siddiqui MS, et al. AASLD Practice Guidance on the clinical assessment and management of nonalcoholic fatty liver disease. Hepatology 2023 77 1797–1835. (10.1097/hep.0000000000000323) 36727674 PMC10735173

