# Supplementary material for: Serum insulin-like growth factor binding protein 2 is associated with hepatic steatosis in adults with metabolic dysfunction-associated steatotic liver disease
Source: Endocr Connect. 2025 Jul 17;14(7):e250285. doi: 10.1530/EC-25-0285 (PMC12278360; doi:10.1530/EC-25-0285)
Supplement: Supplementary file 1 [file supplementary_materials.pdf]

Supplementary materials

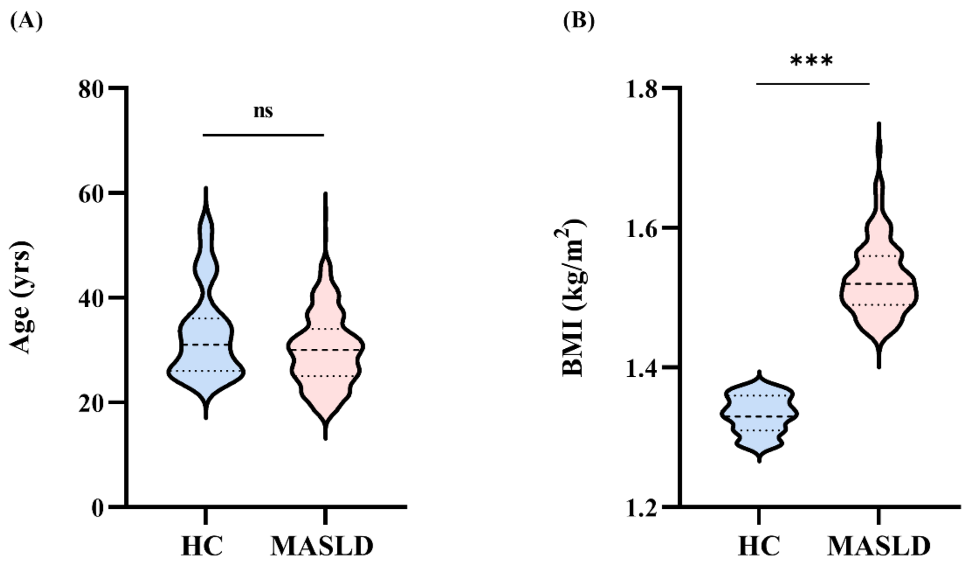

Figure S1

Comparative Analysis of Age and BMI between Healthy Controls and MASLD Patients.

A. Age, and B, BMI. Statistical analysis using t-tests showed comparable age between MASLD and HC groups, whereas BMI was significantly higher in MASLD group. The parameter IGFBP2 with a skewed distribution underwent log(x) transformation to achieve a normal distribution before analysis. $^{***}p < 0.001$; ns = non-significant ($p \geqq 0.05$).

Table S1 Associations of CAP and LSM with serum IGFBP2 levels in the BMI < 40 kg/m^2 cohort (n = 316).

	**Model 1**		**Model 2**	
	***r* value**	***p* value**	***r* value**	***p* value**
CAP (dB/m)	-0.109	0.052	-0.114	0.044
LSM (kPa)	0.057	0.313	0.079	0.164

Note: Correlation analyses were conducted to examine association between CAP, LSM and IGFBP2. Model 1: No variable adjustment (Pearson); Model 2: Adjusted for age, height, and weight based on Model 1 (Partial). The parameter IGFBP2 and LSM with a skewed distribution underwent log(x) transformation to achieve a normal distribution before analysis. *Significance, $p < 0.05$.

Table S2 Associations of serum IGFBP2, body composition, and metabolic variables with CAP in the BMI < 40 kg/m^2 cohort (n = 316).

**Variables**	***B***	***β***	***p value***	**Adjusted R^2**	***F***
IGFBP2 (ng/ml)	-15.189	-0.103	0.048		
BFM (kg)	1.163	0.230	< 0.001		
ALT (U/L)	0.056	0.127	0.020		
TG (mmol/L)	3.525	0.171	0.002	0.168	10.070***
HDL-C (mmol/L)	-10.829	-0.066	0.214		
FINS (mU/L)	0.300	0.089	0.120		
UA (umol/L)	0.039	0.122	0.024		

Note: Multiple linear regression analysis between CAP and possible variables. The parameter IGFBP2 was log-transformed before analysis. $^{***}p < 0.001$.
